# Cause or Coincidence: A Case of Necrotizing Pancreatitis in the Setting of Herpes Simplex Virus Infection

**DOI:** 10.7759/cureus.51028

**Published:** 2023-12-24

**Authors:** Mohamed A Ebrahim, Eli A Zaher, Parth Patel, Muhammad Sohaib Alvi

**Affiliations:** 1 Internal Medicine, Ascension Saint Joseph Hospital, Chicago, USA

**Keywords:** hepatitis, gallstones, necrosis, pancreatitis, hsv

## Abstract

This study explores a rare occurrence of acute pancreatitis induced by herpes simplex virus (HSV) in an immunocompetent adult. The patient, initially diagnosed with pancreatitis presumed to be gallstone-related, exhibited persistent symptoms and elevated lipase levels. Endoscopic ultrasound revealed necrotizing pancreatitis without stones, prompting suspicion of an atypical cause. Subsequent serology confirmed acute HSV infection. This case underscores the importance of considering viral etiologies in atypical pancreatitis cases, especially when hepatitis coexists. The study contributes to the limited literature on HSV-induced pancreatitis in immunocompetent individuals, emphasizing the significance of early recognition and appropriate management in the absence of typical risk factors.

## Introduction

Acute pancreatitis is a common condition with a mortality rate of 1-5%. It mainly presents as abdominal pain, nausea, and vomiting. The majority of cases are caused by gallstones, alcohol, and hypertriglyceridemia. Less common causes include medications, trauma, hypercalcemia, scorpion bites, anatomical variants (e.g., pancreas divisum), and endoscopic retrograde cholangiopancreatography (ERCP) [1]. Infectious causes are rare and mostly limited to viruses, such as mumps, cytomegalovirus, coxsackie B virus, human immunodeficiency virus, Epstein-Barr virus, and severe acute respiratory syndrome coronavirus 2 [1,2]. Herpes simplex virus (HSV) is the least common viral cause of acute pancreatitis, with only one published case in an immunocompetent adult [2].

## Case presentation

A 55-year-old female presented to the emergency room due to sudden onset sharp epigastric pain associated with multiple bouts of nonbloody and nonbilious emesis. No fever was reported. She denied the use of any medications, alcohol, or illicit substances. Her medical history included type 2 diabetes and hypertension without prior admissions or similar symptoms.

**Figure 1:**
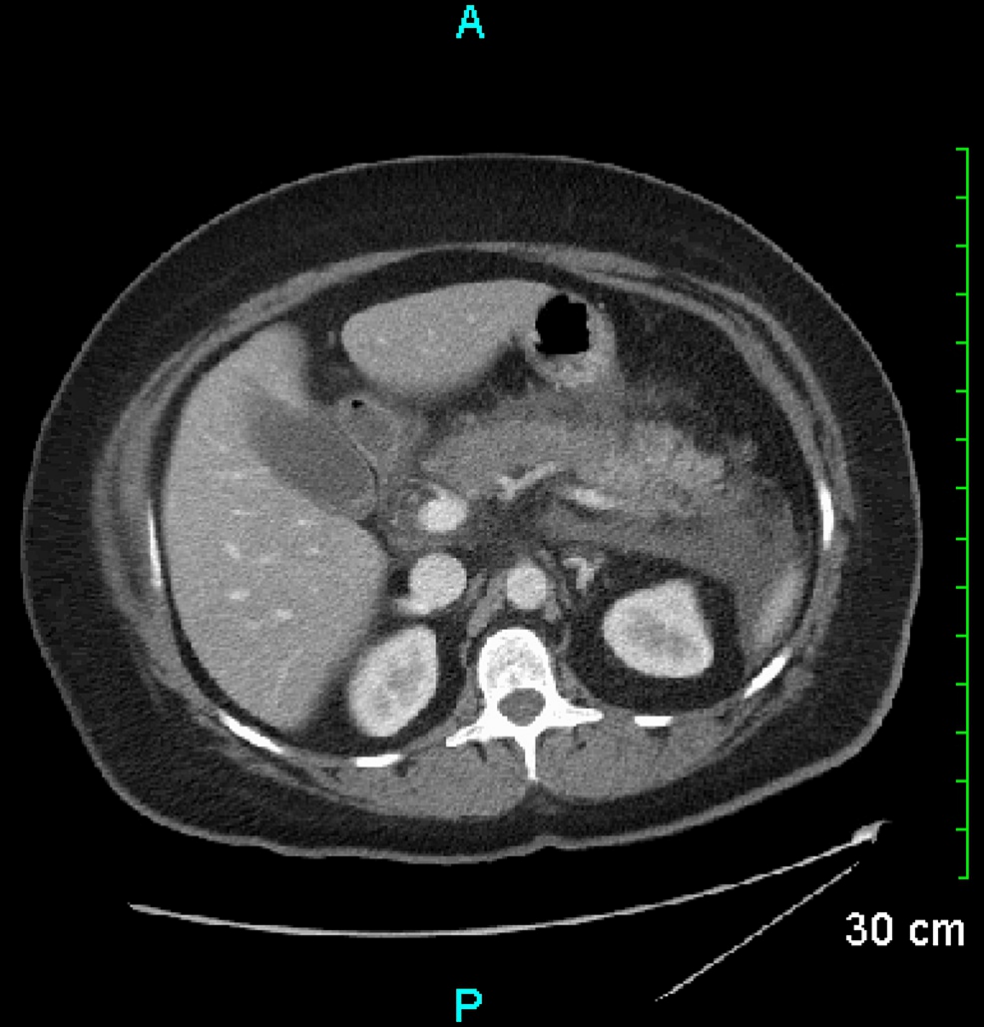
CT abdomen with IV contrast upon admission. Decreased attenuation of the pancreatic body with fat stranding consistent with acute pancreatitis. Gallstones are likewise seen.

On admission, she appeared in distress and her abdominal examination was positive for distention and tenderness in all four quadrants with guarding. Vital signs were within normal limits. Significant labs were as follows: lipase >3,000 IU/L (reference range: 11-82 IU/L), aspartate aminotransferase 482 IU/L (reference range: 13-39 IU/L), alanine aminotransferase 433 IU/L (reference range: 7-52 IU/L), alkaline phosphatase 157 IU/L (reference range: 35-104 IU/L), total bilirubin 3.0 mg/dL (reference range: 0-1.0 mg/dL), triglycerides 121 mg/dL (reference range: 0-150 mg/dL), white blood cell count 28.5 k/µL (reference range: 4.0-11.0 k/µL), calcium 10.4 mg/dL (reference range: 8.5-10.5 mg/dL), and blood alcohol 0 mg/dL (reference range: 0-9 mg/dL). Computed tomography (CT) of the abdomen was consistent with acute pancreatitis and further showed steatosis of the liver and cholelithiasis (Figure 1).

Taken together, she was diagnosed with acute pancreatitis presumably from gallstones. Initial management included bowel rest, intravenous hydration, pain control, prophylactic piperacillin-tazobactam, and a plan for ERCP with subsequent laparoscopic cholecystectomy.

**Figure 2:**
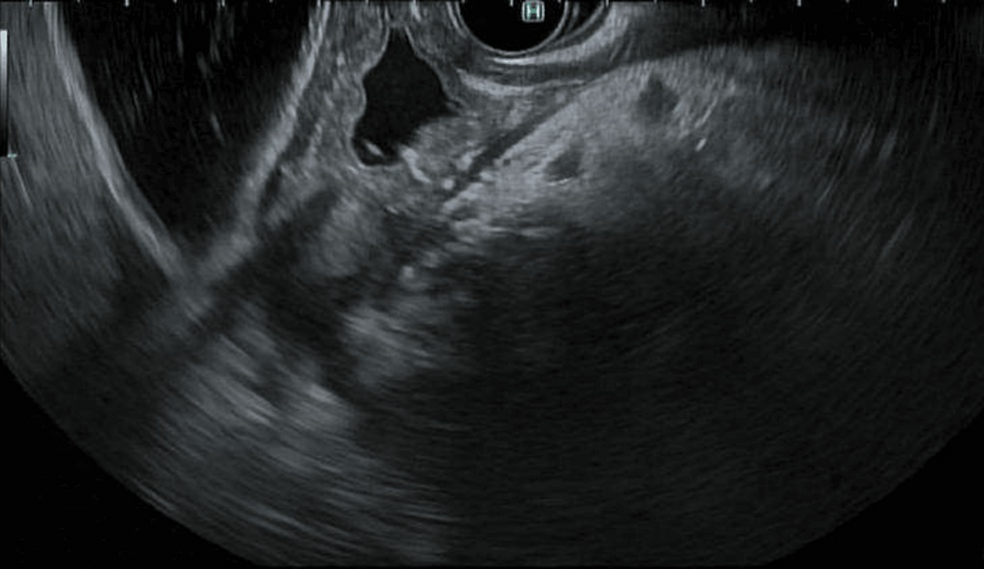
Pancreatic head with a hypoechoic area concerning necrosis without evidence of stones in the pancreatic duct.

**Figure 3:**
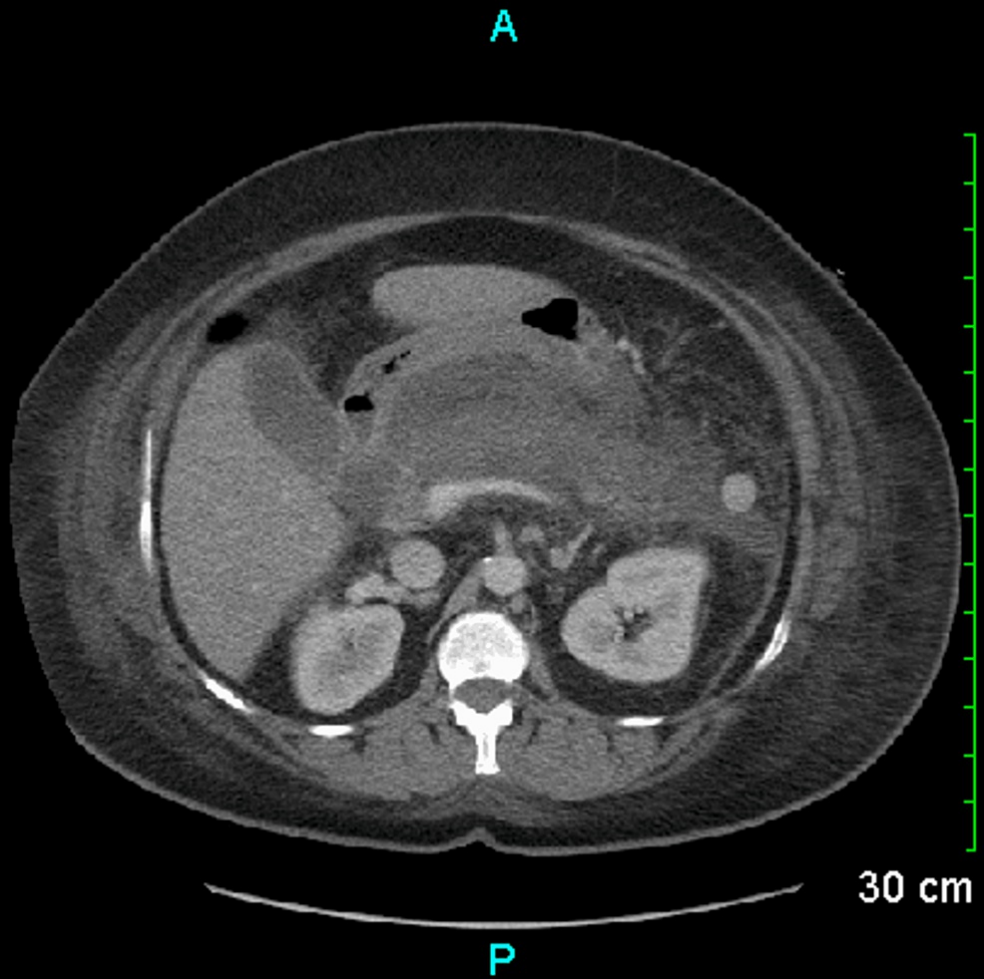
Follow-up CT abdomen on day six of admission. Pancreas with minimal enhancement, concerning necrosis.

Her clinical course was characterized by continuous severe abdominal pain with vomiting and persistently elevated lipase levels, raising suspicion of a stone in the pancreatic duct. An endoscopic ultrasound was hence performed and demonstrated necrotizing pancreatitis without stones (Figures 2, 3). She likewise developed new tender lip ulcers, and treatment with intravenous acyclovir was begun. Subsequent serology was positive for acute HSV infection.

In the following days, her abdominal pain and vomiting began subsiding and she was able to tolerate oral intake. Lab results, including lipase and liver enzymes, also began trending towards normal limits. Acyclovir was then switched to oral and she was discharged home in good health with a plan for outpatient cholecystectomy.

## Discussion

Acute pancreatitis is the most common gastroenterological disease requiring admission to the hospital with an annual incidence of 34 per 100,000 persons in high-income countries [3]. It is considered both local and systemic inflammatory responses involving the pancreas with extension to the peripancreatic tissue. Most patients present with milder forms which self-resolve within one week, while approximately 20% of the patients develop moderate or severe pancreatitis accompanied by necrosis of the pancreatic or peripancreatic tissue which could lead to organ failure and has a mortality rate of 20-40% [4]. Acute pancreatitis is diagnosed by meeting two out of three following criteria: clinical (upper abdominal pain), laboratory (serum amylase or lipase greater than three times the upper limit of normal), and/or imaging that would meet CT, MRI, or ultrasonographic criteria. The current guidelines endorse the identification of the possible cause of the disease as early as possible to aid in preventing any subsequent attack [5]. The most common causes of acute pancreatitis in adults are gallstones and alcohol use, reflecting 45% and 20%, respectively, in most high-income countries. Other etiologies include medication-induced, ERCP, hypercalcemia, hypertriglyceridemia, autoimmune disease, trauma, and infection (bacterial, viral, or parasitic) [6].

Herpes simplex virus is a double-stranded, enveloped DNA virus that induces infection of the mucosal surfaces and rarely leads to disseminated disease, especially in immunocompetent hosts where it usually yields an asymptomatic or mild clinical course. As it is usually transmitted to individuals through infected oral fluids exchanged during close contact, the period between oral infection and symptom onset varies between one and 26 days, typically lasting six to eight days, while the resulting lesion typically lasts for one to eight days. Until now, it is one of the most unusual viral causes of acute pancreatitis [7]. In the literature, four pertinent publications exist. The initial two reports detail three instances of viral pancreatitis linked to HSV [8,9]. However, these cases involve individuals with significant additional comorbidities, such as allergic granulomatous angiitis, invasive pulmonary aspergillosis, and HIV infection. The third report describes a case of acute idiopathic pancreatitis where HSV was isolated from the stomach contents, yet no seroconversion occurred [10]. The last case report simulates this presentation through which a patient without any comorbidities has a high possibility of acute pancreatitis induced by HSV.

Throughout the patient's hospital stay, the clinical observation and laboratory results indicated a cholestatic syndrome as the likely reason behind the pancreatitis, potentially linked to gallstones. However, the additional finding of hepatitis, absence of a stone on endoscopic ultrasound, complicated course of necrosis, and the presence of lower tender lip ulcers pointed to the possibility of a viral cause to her condition. HSV-induced hepatitis is frequently observed and typically remains asymptomatic during the primary infection in adults. However, symptoms might emerge, particularly in cases of a weakened immune system or during pregnancy. Early signs of the disease include fever, fatigue, increased levels of aspartate aminotransferase and alanine aminotransferase in the blood, and leukopenia, often without the occurrence of jaundice [11]. Although herpetic hepatitis is a common condition, its coexistence with pancreatitis is rare. As far as we know, there has been an additional documented instance of a herpes infection impacting both the liver and pancreas, associated with the spread from initial genital herpes during pregnancy [2]. 

The management of herpes simplex virus infections, encompassing both mild and disseminated forms, involves tailored approaches, particularly in immunocompromised patients. Mild cases often benefit from antiviral medications orally, such as acyclovir (400 mg orally three times per day for 7-10 days), valacyclovir (1 g orally two times per day for 7-10 days), or famciclovir (250 mg orally three times per day for 7-10 days) to reduce symptoms and fasten healing. The treatment duration may be prolonged if the healing process remains incomplete following a 10-day therapy period. Conversely, disseminated HSV infections necessitate more aggressive interventions, potentially requiring hospitalization for high-dose intravenous acyclovir therapy (5-10 mg/kg body weight IV every 8 hours), along with supportive care and close monitoring. Treatment should commence before confirmation by serology or polymerase chain reaction (PCR). Preventive strategies, such as safe practices to minimize transmission, antiviral suppression for recurrent outbreaks, and educating patients about the condition, stay essential across all levels of HSV management [12].

## Conclusions

Viral causes of acute pancreatitis are fairly uncommon in immunocompetent adults, with HSV being the most infrequent culprit. It is important to consider viral causes in individuals without typical risk factors for pancreatitis, especially when hepatitis is coexistent. HSV should be suspected in the presence of classic oral lesions, and treatment is warranted prior to serologic or polymerase chain reaction (PCR) confirmation.
